# Conus branch artery utilization in percutaneous coronary intervention for chronic total occlusion

**DOI:** 10.1038/s41598-022-10984-5

**Published:** 2022-05-04

**Authors:** Shih-Wei Meng, Ching-Chang Huang, Chih-Kuo Lee, Chun-Kai Chen, Chih-Fan Yeh, Ying-Hsien Chen, Mao-Shin Lin, Hsien-Li Kao

**Affiliations:** 1 Department of Internal Medicine, National Taiwan University Hospital, Hsin-Chu Branch, Hsinchu, Taiwan; 2 Department of Internal Medicine, National Taiwan University Hospital, 7 Chung-Shan South Rd, Taipei, 10002 Taiwan

**Keywords:** Interventional cardiology, Cardiovascular diseases

## Abstract

Data on the prevalence of conus branch artery (CBA) is scarce, and its utilization in percutaneous coronary intervention (PCI) for chronic total occlusion (CTO) is non-existing. The present study examined carefully in a large cohort the angiographic prevalence of CBA, its role as a collateral channel for the occlusion, and the potential usage of CBA in contemporary CTO PCI. We retrospectively examined consecutive CTO PCIs from our database between 2016 and 2019. All CTO PCIs were evaluated and the results with complications were recorded to determine the prevalence and utilization of CBA. From January 2016 to December 2019, a total of 556 CTO PCI attempts in 546 patients by high-volume operators were enrolled. The clinical, angiographic, and procedural details were collected. CBA was identifiable in 85.3% of these patients, and CBA providing visible collaterals connected to CTO distal lumen was found in 27.8% of patients. 84 CBA were used for balloon anchoring, 17 for selective distal true lumen visualization, and 9 as actual retrograde interventional collateral channel during CTO PCI. Only 1 patient suffered from chest pain during CBA balloon anchoring, and no other procedural complication such as arrhythmia or perforation occurred.CBA is frequently seen in coronary CTO. Its existence provided potential for various CTO PCI technique applications, without increase in risk.

## Introduction

Chronic total occlusions (CTO) is the most challenging lesion subset for percutaneous coronary intervention (PCI), and may compose 20% of all patients referred for diagnostic coronary angiography in daily practice^1,2^. Due to the improvement of devices and experience, the successful rate of CTO PCI improved significantly^3,4^. Retrograde approach is an integral part in the contemporary CTO PCI algorithm^5–7^. Careful examination and selection of the feasible collateral channel is very important in retrograde approach, and certain scoring systems had been established^8–10^. However, previous studies on septal and epicardial channels rarely mention the unique and ubiquitous conus branch artery (CBA). We described hereafter an easily overlooked but frequently existing vessel, CBA, of its prevalence and usefulness in CTO PCI.

## Methods

We retrospectively examined the coronary angiography of our CTO PCI database from January 2016 to December 2019. The procedures were all done by high-volume operators, defined as > 75 total CTO PCI cases and > 20 retrograde attempts during the study period, proposed by Thompson et al.^11^. Coronary CTO was defined as a totally occluded segment with Thrombolysis in Myocardial Infarction flow grade 0, and an estimated duration of at least 3 months. The basic characteristics of patient, including age, sex, renal function, etc. as well as the angiographic data and intervention details were obtained and analyzed.

Pre-specified characterization of CBA was coded by 2 independent interventionists, with the following items recorded: (1) the presence of CBA, (2) if CBA diameter is ≥ 1.5 mm in diameter, (3) CBA collateral connection to the true lumen distal to CTO segment, and (4) the actual CBA collateral utilization during CTO PCI. The retrospective review of the clinical information and radiologic records of the patients was approved by Institutional Review Board of National Taiwan University Hospital with the proof number 201612048RINA. The study was performed in accordance with relevant guidelines and regulations. As a retrospective study, the need of informed consent was waived by the Institutional Review Board of National Taiwan University Hospital.

### Statistical analysis

Analysis was performed using SPSS version 25 for Windows (SPSS Inc., IL, USA). Continuous data was expressed as mean ± standard deviation. Categorical data were expressed as number and percentage (%). Differences between proportions were calculated using the chi-square test or Fisher’s exact test. Comparisons of data between 2 groups were performed using the independent T test (normally distributed data). A two-sided *P* value less than 0.05 was defined as statistically significant.

## Results

A total of 584 CTOs in 574 patients were attempted. After excluding patients with missing data or angiography, 546 patients with 556 CTO were finally included in this analysis. Most patients were male (n = 481, 88%), with the mean age of 62.8 ± 11.4 years-old. Of all the 556 attempted CTO, right coronary artery (RCA) was the most common target vessel (n = 277, 50%), followed by left anterior descending artery (LAD) (n = 207, 37%). All procedures were transfemoral. A 7 Fr Terumo sheath was used as the default size of the vascular access for primary strategy (either antegrade or retrograde), while another 6 Fr sheath was inserted at opposite side if necessary. At the end of the procedure, angiography of femoral artery was routinely checked to identify access related complications. The overall successful rate of CTO recanalization was 95.9% (533/556). Of all 566 CTOs, 242 (43.5%) was successfully recanalized using antegrade-only approach, 207 (37.2%) using retrograde-only approach, and the rest combined antegrade and retrograde approach. The baseline demographics and target lesions characteristics were summarized in Table 1. Procedural and technical details were summarized in Table 2.

**Table 1:** Demographic and Target Lesion Characteristics.

Per patient (n = 546)	
Age, y, (mean ± SD)	62.8 ± 11.4
Male sex (%)	481 (88.1%)
Hypertension (%)	398 (72.9%)
Diabetes mellitus (%)	206 (37.7%)
Hyperlipidemia (%)	332 (60.8%)
Smoking (%)	253 (46.3%)
Per CTOs (n = 556)	
LAD CTO	207 (37.2%)
LCX CTO	66 (11.9%)
RCA CTO	277 (49.8%)
LM CTO	2 (0.36%)
Dx CTO	3 (0.54%)
OM CTO	1 (0.18%)
J-CTO score	2.87 ± 1.21

*LAD* left anterior descending artery, *LCX* left circumflex artery, *RCA* right coronary artery, *LM* left main coronary artery, *Dx* diagonal branch, *OM* obtuse marginal branch, *CTO* chronic total occlusion.

**Table 2:** Procedure Outcomes and Technical Parameters.

Target (n)	Success (%)	Fluoroscopy time (minutes)	Procedure time (minutes)	Contrast volume (mL)
LAD (207)	202 (97.6)	41.52 ± 25.31	87.71 ± 46.86	277.16 ± 103.12
LCX (66)	62 (93.9)	45.92 ± 31.02	93.83 ± 53.35	252.12 ± 117.15
RCA (277)	263 (94.9)	50.74 ± 27.85	100.87 ± 49.74	245.61 ± 101.60
LM (2)	2 (100)	20.5 ± 0.5	60 ± 0	300 ± 50
Dx (3)	3 (100)	42.67 ± 38.44	73.33 ± 61.28	143.33 ± 91.04
OM (1)	1 (100)	44	120	300
Total (556)	533 (95.9)	46.57 ± 27.71	94.87 ± 49.53	257.87 ± 105.27

*LAD* left anterior descending artery, *LCX* left circumflex artery, *RCA* right coronary artery, *LM* left main coronary artery, *Dx* diagonal branch, *OM* obtuse marginal branch.

**Figure 1:**
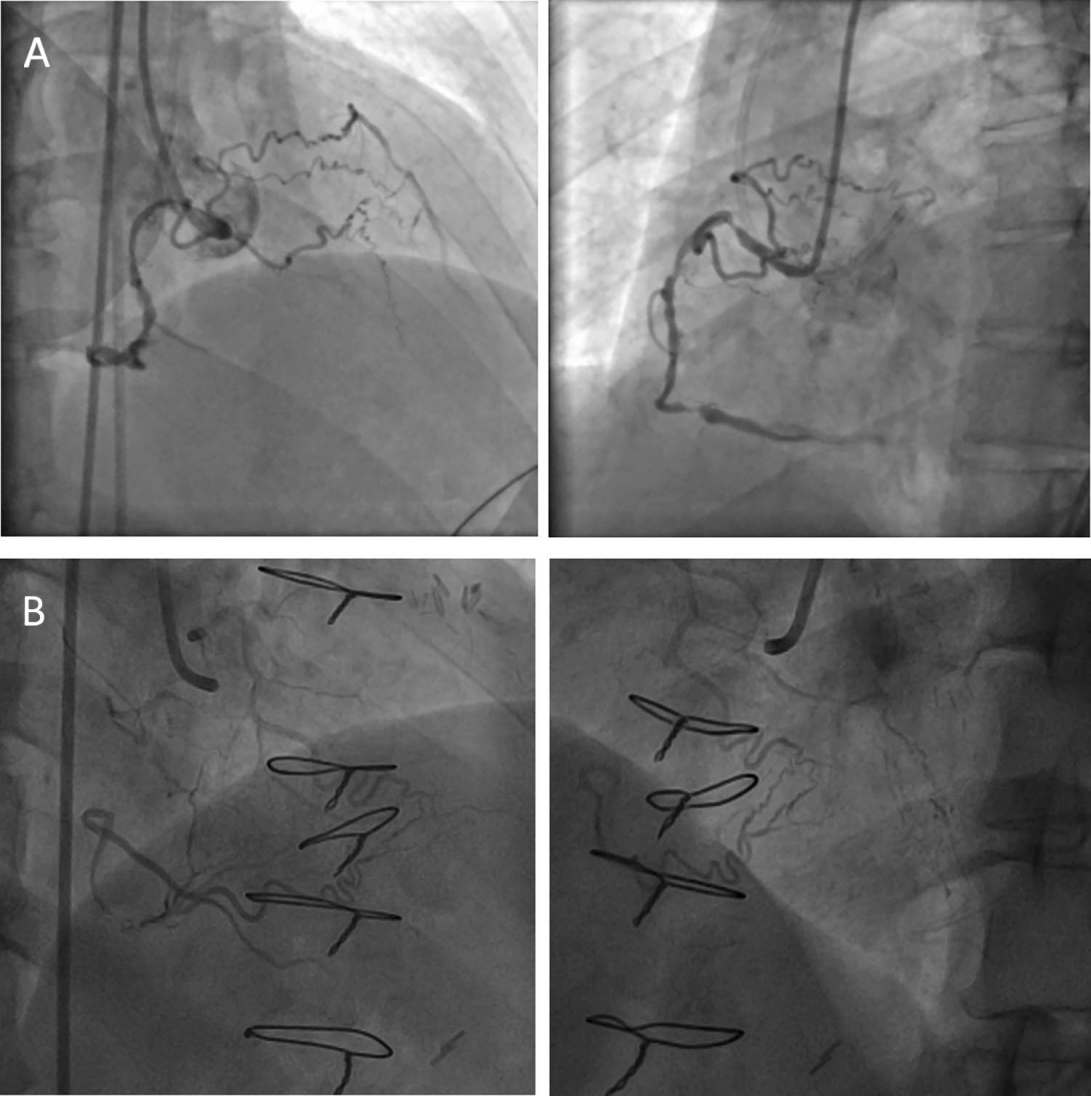
(**A**) Right anterior oblique and left anterior oblique projections of a conus branch artery collateral to left anterior descending artery. (**B**) Right anterior oblique projection of a conus branch artery collateral to distal RCA via a tortuous channel.

**Figure 2:**
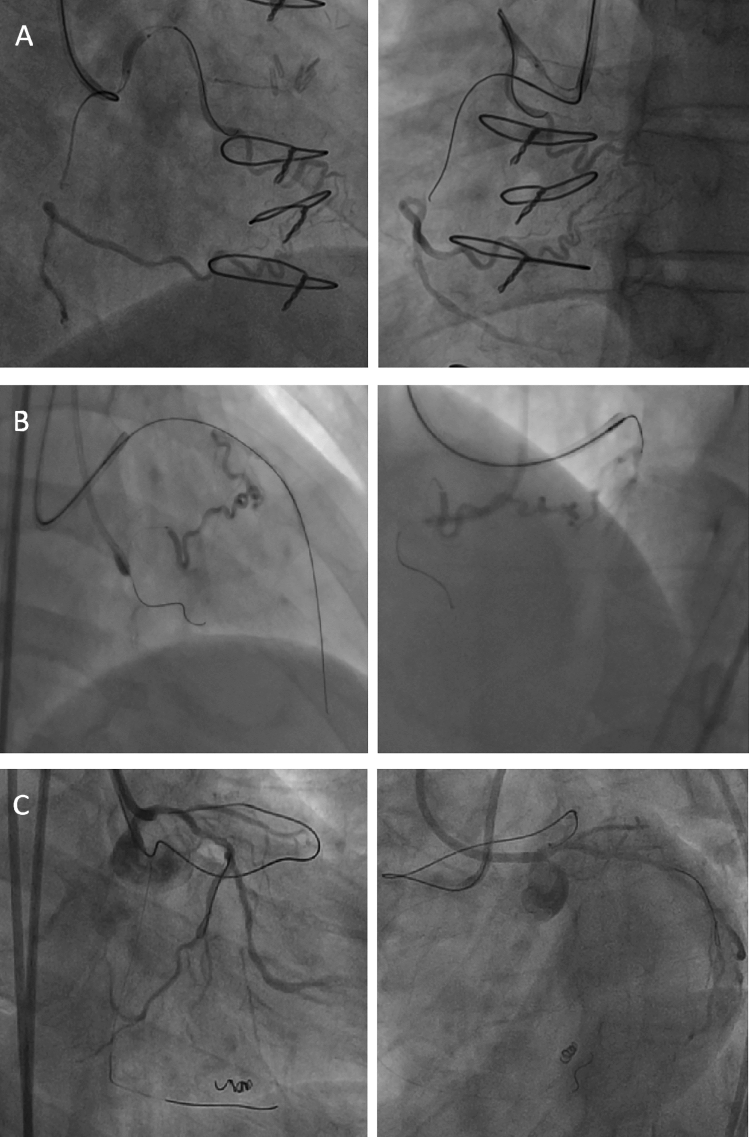
Utilization of conus branch. (**A**) balloon anchor to stabilize guiding catheter, (**B**) selective injection for antegrade wiring, (**C**) serve as retrograde collateral channel.

The presence of a CBA with diameter ≥ 1.5 mm was high (70.5%, 385/546, per patient) in this study population. CBA providing collateral connection to the distal true vessel could be found in about one-fourth (27.3%, 152/556) overall, which could potentially be used for distal lumen opacification or serve as collateral channel for retrograde approach. These 152 CBA connections included 94 for the 207 LAD CTOs (45.4%, 94/207), 57 for the 277 RCA CTOs (20.6%, 57/277), and another 1 for left main coronary artery CTO. There was no CBA connection for the LCX CTOs. In the actual procedure, CBA was utilized for balloon anchoring to stabilize guiding catheter in 84 cases, for microcatheter (MC) tip selective injection facilitating antegrade wiring in 17, and tracked as collateral channel for retrograde approach in 9. The existence and utilization of CBA were listed in Table 3 according to the target CTO. Table 4 showed the procedure details stratified by CBA utilization and target vessels. The J-CTO score were 2.80 ± 1.23 and 3.10 ± 1.21 in groups not using and using CBA, respectively (*p* = 0.028, T test). The successful rate of CTO intervention in groups not using and using CBA were 95.68% and 95.23%, respectively (p = 0.791, Fisher’s exact test). Figure 1 depicted typical CBA serving as collateral to distal true vessel, in LAD and RCA CTO respectively. Figure 2 illustrated different actual usages of CBA during CTO intervention.

**Table 3:** Conus Branch Existence and Utilization During CTO PCI (per CTO).

Target (n)	Conus branch existence (%)	Conus branch ≥ 1.5 mm (%)	Connection to distal true lumen (%)	Balloon anchor (n)	Selective MC tip injection (n)	Retrograde collateral channel tracking (n)
LAD (207)	183 (88.4)	153 (83.6)	94 (51.4)	5	16	7
LCX (66)	52 (78.8)	35 (67.3)	0	1	0	0
RCA (277)	237 (85.6)	194 (81.9)	57 (24.1)	78	1	2
LM (2)	1 (50)	1 (100)	1 (100)	0	0	0
Dx (3)	3 (100)	2 (66.7)	0	0	0	0
OM (1)	0 (0)	0	0	0	0	0
Total (556)	476	385 (80.9%)	152 (31.9%)	84	17	9

*LAD* left anterior descending artery, *LCX* left circumflex artery, *RCA* right coronary artery, *LM* left main coronary artery, *Dx* diagonal branch, *OM* obtuse marginal branch, *MC* microcatheter.

**Table 4:** Procedure Details Stratified by Utilization CBA and Target Vessel.

	J-CTO score	Success (%)	Fluoroscopy time (minutes)	Procedure time (minutes)	Contrast volume (mL)
Conus existence with utilization (n = 105)	3.10 ± 1.21*	100 (95.23)^+^	55.24 ± 31.16*	108.91 ± 54.98*	263.81 ± 89.54^+^
LAD (n = 25)		23	58.64 ± 41.42	111.60 ± 72.47	305.6 ± 87.51
LCX (n = 1)		1	31	60	230
RCA (n = 79)		76	54.47 ± 27.48	107.10 ± 48.60	251.01 ± 87.14
Conus existence without utilization (n = 371)	2.80 ± 1.23*	355 (95.68)^+^	44.84 ± 26.48*	91.75 ± 47.58*	257.72 ± 108.70^+^
LM (n = 1)		1	21	60	350
LAD (n = 158)		155	39.58 ± 21.33	84.07 ± 41.57	276.09 ± 102.08
Diagonal (n = 3)		3	42.67 ± 47.08	73.33 ± 75.06	143.33 ± 111.50
LCX (n = 51)		47	46.65 ± 32.13	93.88 ± 52.89	246.96 ± 126.92
RCA (n = 158)		149	49.70 ± 27.96	99.29 ± 50.14	244.40 ± 106.20

*LAD* left anterior descending artery, *LCX* left circumflex artery, *RCA* right coronary artery, *LM* left main coronary artery.

*p < 0.05, ^+^*p* > 0.05, between with and without utilization groups.

The complication rate of the whole series was low (0.9%, 5/556), including 1 retroperitoneal hematoma managed conservatively, 1 femoral artery bleeding treated with stent graft, 1 collateral channel (AV groove) extravasation treated with coil embolization, 1 septal hematoma caused by MC stretching during retrograde approach managed conservatively, and 1 donor vessel thrombosis during retrograde approach requiring emergent bypass surgery. There was no clinically significant complication specific to the utilization of CBA. 1 patient complained of intolerable chest pain during balloon anchoring in CBA. We did not record complete ECG during the episode, but there was no obvious ST change on the monitor leads. His symptoms resolved after deflating the anchored balloon, without any subsequent event.

## Discussions

CBA supplies blood flow to the conus, or outflow tract, of the right ventricle and is generally considered to be the first branch of RCA. On coronary angiography, CBA is best viewed in the left anterior oblique (LAO) and right anterior oblique (RAO) projections. CBA should be ubiquitous, in theory, and CBA ≥ 1.5 mm in diameter could be found in approximately 90% in our cohort. A prior prospective angiographic study also showed 80.5% incidence of CBA^12^, and the percentage of a CBA originating from an independent ostium may range from 10 to 50% in the literature^13^. As CBA takes off early from RCA, and may even have an independent ostium in the right sinus of Valsalva, it can be easily missed if the catheter was engaged deeply into RCA. Careful positioning of the diagnostic catheter and a high awareness of its independent take-off are important in identifying CBA. Existence of CBA connections to distal vessel segments could be found in 27.3% of CTOs in our present series. However, CBA collaterals were found in only 2.6% (14/519, including 5 in RCA and 9 in LAD CTOs) of 519 CTOs examined in a recent retrospective study^14^. This discrepancy highlighted the importance of adequate and thorough diagnostic imaging and reviewing skills.

The existence of CBA may facilitate CTO PCI in many aspects. First, it may serve as a vessel for wire/balloon anchoring, to stabilize and provide back-up support for RCA guiding catheter. In our series, this practice was used in 15.1% (84/556) of the CTO PCI. Secondly, CBA may provide collateral circulation to the true lumen distal to the CTO. For mid RCA occlusion, CBA may collateralize distal RCA via epicardial connection to right ventricular branches. For proximal or mid LAD occlusion, CBA may collateralize distal LAD via the vascular ring of Vieussens^14^. In the present study, collaterals arising from CBA could be found in 27.3% (152/556) of the CTO, and most frequently in LAD CTO. CBA collateral provides distal true vessel visualization and clear target guidance for antegrade wiring. When a MC was placed in CBA collateral for selective injection, the contrast usage may be reduced significantly. In the present study, actual placement of MC in the CBA for antegrade wiring target visualization was done in 3% (17/556). Lastly, CBA collateral can even be used as an interventional channel for retrograde approach. This practice was applied in only 1.4% (8/556) in the present series, but may increase in the future with the continuous improvement of guide wire and microcatheter.

Strong back-up guiding catheter is usually chosen in CTO PCI, and usually results in deep engagement of coronary ostium, especially the RCA. This may interfere or even prevent utilization of CBA, as the percentage of a CBA with an independent ostium or early take-off is high^12,13^. In our practice, we usually use less selective guiding catheters, such as JR4, with balloon anchor if necessary. This practice provides strong backup similar to that with a supportive catheter, and mitigates possible trauma or dissection by catheter deep engagement.

As we mentioned earlier, the successful rates of CTO interventions with or without CBA utilization were both above 95% without statistical difference. However, in the CBA utilization group, the J-CTO score was statistically higher. Moreover, in the CBA utilization group, more retrograde technique was applied (65.7% vs. 54.2%, *p* = 0.035, chi-square test). We also found longer fluoroscopy time (55.24 ± 31.16 vs. 44.84 ± 26.48 min, *p* = 0.001) and procedure time (108.91 ± 54.98 vs. 91.75 ± 47.58 min, *p* = 0.002) in the CBA utilization group, which might be attributable to more difficult lesions and more retrograde procedures. The contrast volume used between both groups was not statistically different (263.81 ± 89.54 vs. 257.72 ± 108.70 mL, *p* = 0.60). Despite these characteristics suggesting more challenging case scenario in the CBA utilization group, the successful rate was comparable to those without. This may suggest the importance of CBA utilization in facilitating certain complex CTO PCI. On the other hand, since no difference in success rates could be found in both groups, the roles and timing of CBA utilization in CTO interventions should be further investigated in future prospective study.

During CTO PCI, procedure complications are always a concern. Major events including death, Q-wave myocardial infarction, donor vessel dissection, equipment entrapment, coronary artery perforations, and emergent cardiac surgery were carefully recorded in our database. In the present cohort, we reported a high successful rate but a low complication rate, compared to prior studies^3,15^. The complication rate was also lower compared to our prior study^10^. Possible explanations included improvement of devices, along with the increasing experience and skills of CTO PCI.

Some specific safety considerations when utilizing CBA during CTO PCI warrant discussion. CBA supplies perfusion to the outflow tract of right ventricle, therefore, prolonged balloon inflation for anchoring or microcatheter indwelling for retrograde approach inside CBA may cause conus myocardial ischemia or even life-threatening arrhythmia^16^. There have been prior reports of ST segment elevation during acute ischemia of the right ventricular outflow tract, which are similar to the electrocardiographic (ECG) findings of Brugada syndrome^16,17^, and even ventricular fibrillation induced by iatrogenic CBA spasm^18,19^. In our series, however, only 1 out of 84 patients complained of chest pain during CBA balloon anchoring. None of the other patients in whom their CBAs were used for selective contrast injection (n = 17), or as collateral channel for retrograde approach (n = 8), had any complaint of discomfort. The prolonged interruption of CBA blood flow by anchoring balloon or indwelling microcatheter did not result in any significant ECG changes, arrhythmia, nor hemodynamic instability in our patients.

CBA perforation is another potential catastrophic complication. Rupturing CBA or the collateral channels it supplies will lead to hemopericardium or pericardial tamponade. The resulting hematoma and inflammation may also cause subsequent myocardial damage. Luckily there was no CBA perforation in the present study. Just like treating any epicardial coronary vessels and collaterals, careful application of correct devices and skills in handling CBA during CTO PCI should be emphasized.

## Limitations

There were some limitations. First, this was a retrospective analysis of CTO PCI performed by high-volume operators; hence extrapolation of results should be cautious and may not be universally applicable. Second, this study provided only descriptive statistics of CBA utilization in CTO intervention because of only one cohort without grouping assignment. The case number was relatively small, therefore future prospective multicenter registry with larger case number is needed to validate our findings. Third, despite procedures with CBA utilization were associated with more complex lesions, longer fluoroscopic time, and more retrograde approaches compared with those without, no differences in success rates could be found between groups. The roles and timing of CBA utilization in CTO interventions should be further investigated. Fourth, complete ECG recording, especially the routine precordial V1-V2 leads, was not performed. It is mandatory to document Brugada type ST changes and define its clinical significance during CBA occlusion in the future study.

## Conclusions

The prevalence of CBA ≥ 1.5 mm in diameter is high in patients with coronary artery CTO. CBA provides interventionists various technical possibilities, including anchoring, selective contrast injection, and collateral for actual retrograde approach, to facilitate PCI success without observable immediate complication.

## Abbreviations

CBA: Conus branch artery
CTO: Chronic total occlusion
PCI: Percutaneous coronary intervention
LAD: Left anterior descending artery
LCX: Left circumflex artery
RCA: Right coronary artery
ECG: Electrocardiography
